# Transanal recto-anal anastomosis for treatment of rectal atresia: a review of 4 cases

**DOI:** 10.1186/s12887-023-03859-9

**Published:** 2023-01-28

**Authors:** Sameh Shehata, Mohamed ElSawaf, Mostafa Kotb

**Affiliations:** 1 Department of Pediatric Surgery, Alexandria Faculty of Medicine, Alexandria, 21615 Egypt; 2 Pediatric Surgery, Tanta Faculty of Medicine, Tanta, Egypt

**Keywords:** Rectal atresia, Recto-anal anastomosis, Transanal approach

## Abstract

**Introduction:**

Rectal atresia is a rare subtype of anorectal malformations in which the patients are born with a normal anal canal but have complete atresia located few centimeters proximal to the dentate line. We present the transanal end-to-end rectoanal anastomosis as a surgical technique for the management of these patients, highlight the outcomes, and emphasize on some clinical tips.

**Methods:**

Four patients were diagnosed as having rectal atresia on clinical and radiological basis. All of them underwent single loop low sigmoid colostomy in the first 24 h. After 6 months, transanal end-to-end rectoanal anastomosis was performed followed by closure of the stoma 3 months later.

**Results:**

The 2 cases that are older than 3 years demonstrated normal continence and are clean between bowel movements, while the other two showed good anal tone and passing stools between 1–3 times daily, being dry in between.

**Conclusion:**

Transanal recto-anal anastomosis allows a safer route of anatomical reconstruction of the anorectum, therefore avoiding the potential complications associated with the other more invasive approaches.

## Introduction

Rectal atresia is a rare condition that comprises about 0.3–1.2% of all anorectal malformations [1]. This anomaly composed of a well-developed pelvic structures, i.e. the anal canal, external and internal sphincters are complete. Moreover, the anal canal and part of the rectum are developed; however, they are separated by an atretic segment of rectum [2]. Usually, no fistula between the rectum and the vagina or urethra is present [1, 3].

Previous reports described various approaches and techniques in the management of this rare type. The treatment of rectal atresia is still controversial and the best operation to address this uncommon condition is still a matter of debate. According to the largest reported series of rectal atresia patients, posterior sagittal approach was found a useful technique. Through this approach, exposes the rectal pouch is exposed, mobilized from the surrounding muscle fibers followed by a direct end-to-end anastomosis between the blind tips of the anus [4]. Other techniques reported in literature include magnetic compression anastomosis, Duhamel pull-through, transanal end-to-end rectoanal anastomosis and laparoscopic assisted transanal approach [5].

Ideally, while operating a case of rectal atresia, an ideal operation should preserve the anatomy leaving the pelvic region without scars or damage. We believe that the transanal approach can fulfill the aforementioned requirements and, therefore, provides good fecal continence. The aim of this paper is to present the technique and outcome of the transanal end-to-end recto-anal anastomosis in the management of rectal atresia.

## Patients and methods

**Fig. 1:**
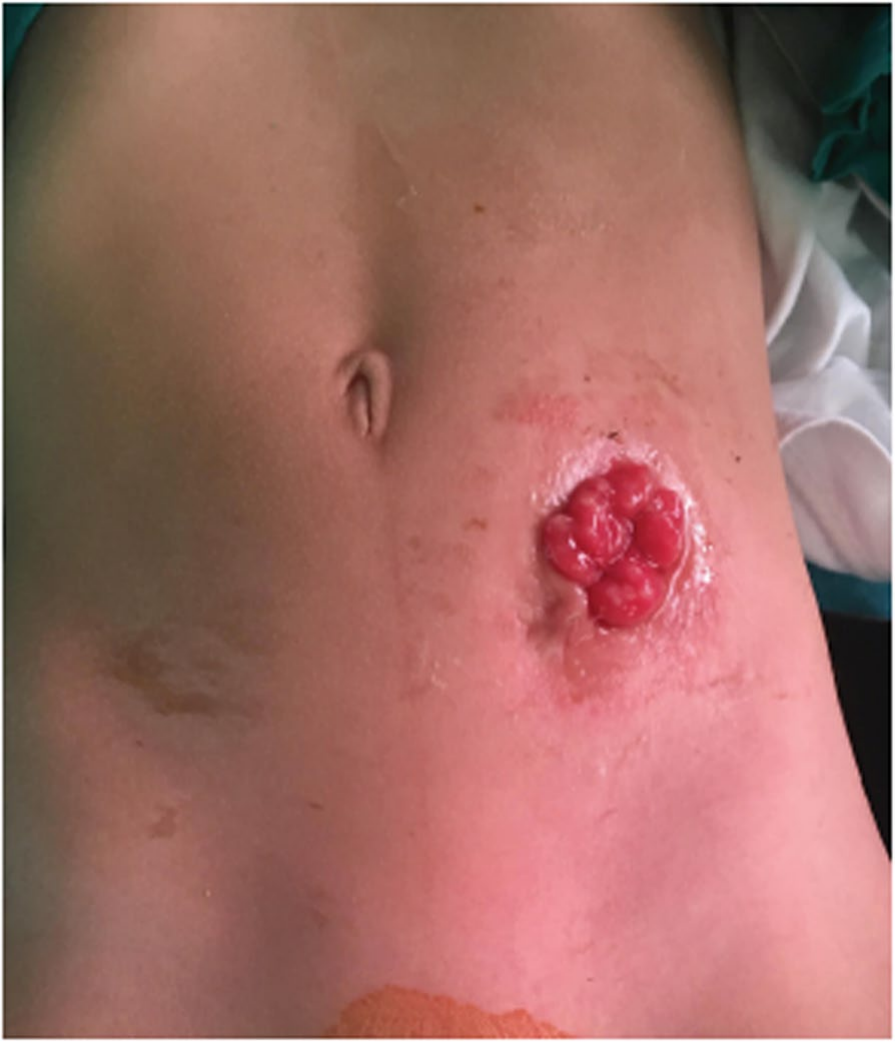
Lower sigmoid simple loop colostomy done in the first 24 h

All patients diagnosed with rectal atresia between 2019 and 2021 were included in our prospective study. Once admitted, the patients’ demographic data were collected. This is followed by thorough history and physical examination including inspection of the abdomen and the pelvic region for external genitalia and the anus as regards its patency and location. Rectal examination with a thermometer to confirm the blind ended anal canal was mandatory. Fistula was checked by observing for meconium either per urethra or via a perineal fistula. Rectal atresia was confirmed only when they failed to pass meconium or when it was found that a thermometer would not pass into the rectum. To check for associated anomalies, echocardiography, ultrasound abdomen and spine were done. For all cases, lower sigmoid simple loop colostomy through an incision in the left lower quadrant was done in the neonatal age (Fig. 1).

**Fig. 2:**
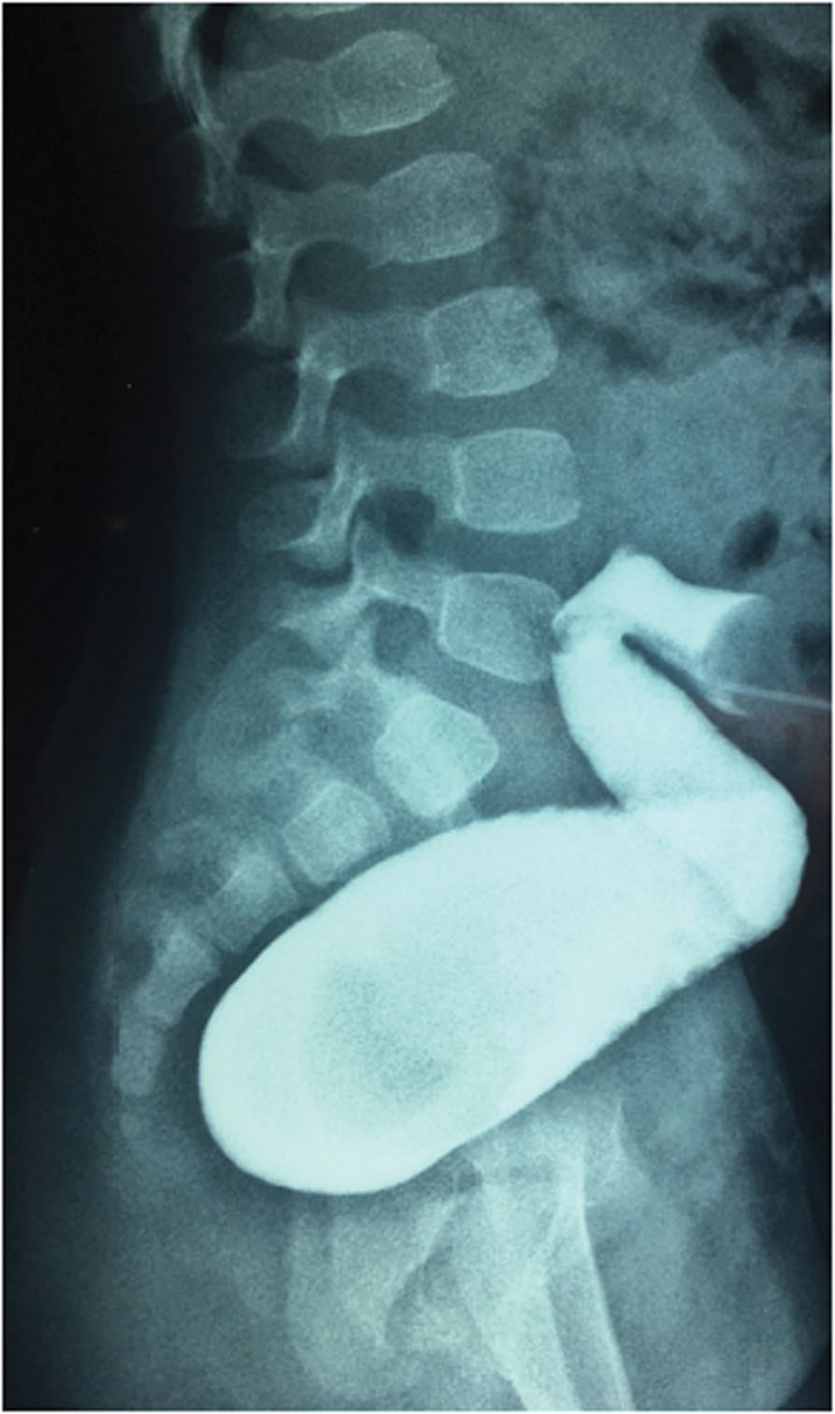
Distal cologram showing the rectal atresia without any fistula

Postoperatively the distal segment of the colostomy was cleaned by an enema from the distal stoma opening once weekly to avoid fecal accumulation and rectal distention. Next, at 4 months of age, a lateral view distal colostogram was carried out to check for the size of the blind ended rectum with its relation to the marked distal rectum as well as the site of possible fistulation (Fig. 2). This is followed by a cystourethrogram before definitive surgery to check for any urinary tract anomalies including urinary fistulas.

**Fig. 3:**
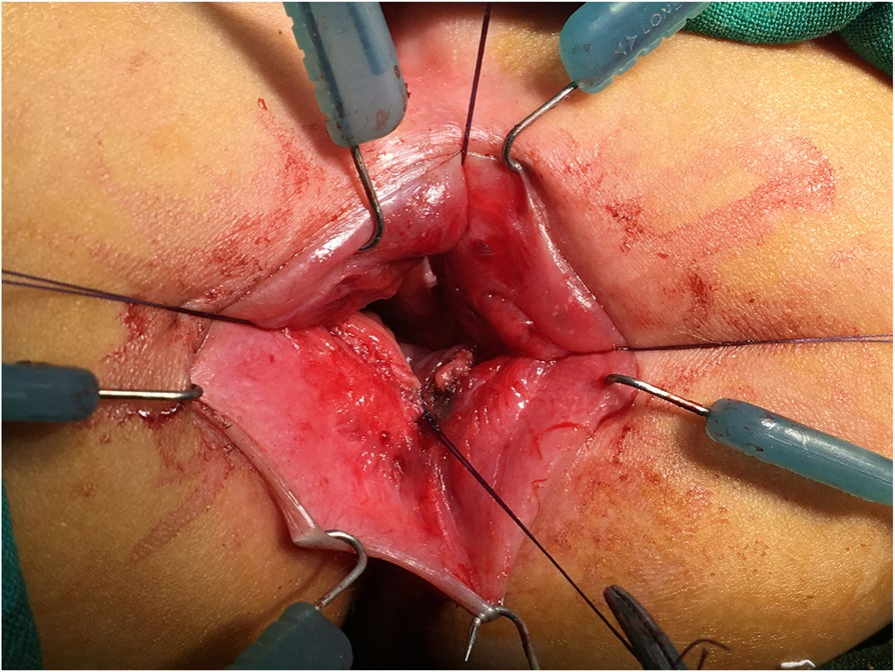
Transanal rectoanal anastomosis above the dentate line

**Fig. 4:**
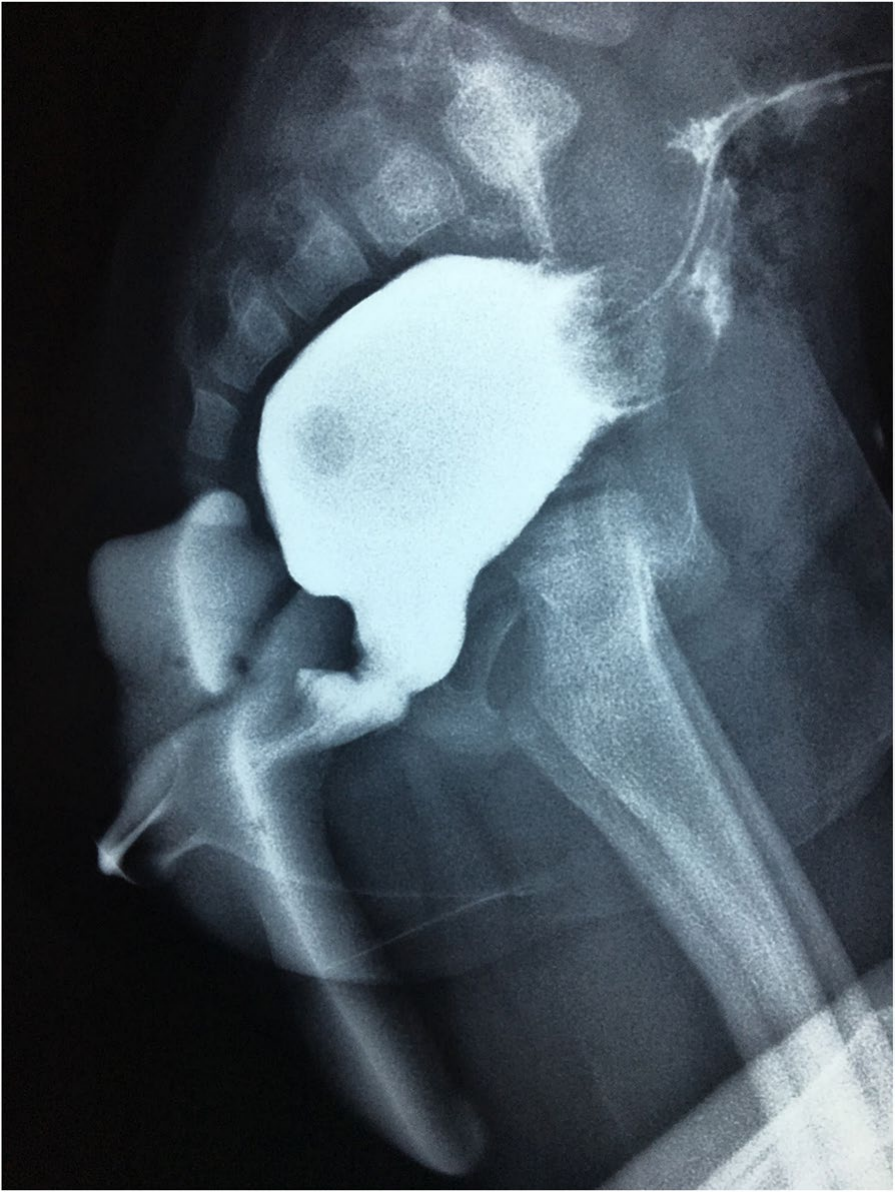
Postoperative contrast study showing absence of stricture at the anastomotic site

The patients were prepared for the definitive surgery 6 months after the colostomy was performed. The patients were operated on using the transanal approach (Fig. 3). After maximal dilatation of the anal canal with Hegar, four full-thickness stay sutures were placed in at 3, 6, 9 and 12 o’clock positions at the anal verge. One of the operating surgeons passed a Hegar dilator through the sigmoidostomy down to the blind end of the rectum with sustained pressure to enable the identification of the bulged upper pouch. A midline incision was made over the tip of the Hegar followed by mobilization of the rectum in the perirectal plane to allow tension-free anastomosis as done in other anorectal malformations. The anastomosis was carried out using delayed absorption sutures in one layer of interrupted sutures. Routine anal dilatation was initiated after 15 days and continued for 1–3 months. Closure of the sigmoidostomy was performed 2 months later; however, a contrast enema was carried out before closure to ensure that there was no stricture (Fig. 4). Follow up was performed after 12 and 24 months for each patient. In each visit, an enquiry about passage of stool and continence was done and the overall Kelly’s score was recorded.

## Results

Four patients, 3 males and 1 female, were referred to us for correction of rectal atresia. By screening for associated conditions, none of them suffered from major congenital anomalies. All of the cases presented with abdominal distention and failure to pass meconium after 24 h of birth. Pelvic examination revealed a normal located patent anus and absent meconium per urethra in all cases. Rectal atresia was confirmed by the failure to pass a thermometer up to the rectum. Lower sigmoid diverting colostomy was performed for all patients within 24 h of admission; none had suffered from post operative complications. Transanal recto-anal anastomosis was done after an average of 6 months. Uneventful post operative course was noticed in the 4 cases. The patients have subsequently had their ostomies reversed, and they have all demonstrated the ability to pass stool per rectum. All patients underwent postoperative anal dilations. The number of dilatations ranged from 1 to 4 with a mean of 2.75 dilatations. None have developed a postoperative stricture. At the time of this report, two cases were older than 3 years, with a median follow up period of 29 months. They have demonstrated normal continence and were clean between bowel movements. The overall Kelly’s score was 5 in both, which is considered a good one (Range: 0–6). On the other hand, the other two showed good anal tone and passing stools between 1–3 times daily, being dry in between.

## Discussion

Rectal atresia is a very rare congenital anomaly with an incidence is of about 1%-2% of all anorectal malformations [2]. It shows marked male predominance with a male: female ratio of 7:1 [6]. Rectal atresia, in contrast to other anorectal malformations, is characterized by a patent and normally located anus and the anal canal and lower rectum are surrounded by a normally developed sphincter, hence a good functional outcome is expected postoperatively [7]. Unlike other anorectal malformations, rectal atresia usually has no associated fistula with the urogenital system [8] as seen in our series. Only few exceptional cases that showed fistula communications, eg. rectourethral [9], rectovestibular [10], and rectolabial fistulae [11].

In the newborn period, workup for associated anomalies is crucial. We recommend echocardiography, ultrasound abdomen and spine. Next, a diverting colostomy should be performed in all cases of rectal atresia. Beside its role as a temporary relief till the age of definitive repair, it offers the advantage of performing distal colostogram to confirm with certainty the absence of any fistulous communication with the urinary tract as well as estimating the distance between the distal rectal pouch and anal canal [12]. However, two precautions are needed to be addressed. First of all, the distal segment of the colostomy needs to be cleaned by an enema from the distal stoma opening once weekly to avoid fecal accumulation and rectal distention in the postoperative period [13]. Secondly, unlike the other anorectal malformations where the site of the stoma is low descending, the recommended site of the stoma should be low sigmoid to enable easier identification of the upper pouch by passing a Hegar dilator or endoscope.

The treatment of rectal atresia is still controversial and several operative procedures have been described, all aimed to maintained optimal continence [14]. This simply reflect the difficulties facing the surgeons in treating this condition, yet they showed their innovative capacity [13]. These include magnetic compression anastomosis [15], transanal endoscopic-assisted proctoplasty (TAEAPP) [3], Duhamel pull-through [16], posterior sagittal anorectoplasty (PSARP) [12], transanal end-to-end rectoanal anastomosis [17] and laparoscopic assisted transanal approach [18]. Since the anal canal and lower rectum are usually well developed and are surrounded by a normal sphincter, the long-term prognosis of these patients is excellent in term of bowel control and continence.

There are several reports, discussing the outcomes of transanal approach for the management of rectal atresia. Upadhyay performed this technique in two cases using Hegar for identification of the blind end of the upper pouch [19]. On the other hand, Stenström et al. used an endoscopy instead in one case and found that the light emitted from the endoscopy gave a better view [13]. Comparing this technique to other techniques, all cases undergoing transanal repair reported normal bowel function after follow-up period ranging from 3–36 months. On the other hand, cases who underwent PSARP showed constipation in around 25% of the cases and occasional soiling in one patient [5].

The transanal end-to-end rectoanal anastomosis approach has many benefits over the standard posterior sagittal approach (PSARP) to repair rectal atresia. Firstly, it enables anatomical anorectal reconstruction under direct visual control rather than extensive anorectal dissection or any division of the sphincter musculature, thus avoiding the potential complications associated with the open posterior sagittal approach. Secondly, it offers the advantage of simultaneous closure of the sigmoidostomy [3], though this was not done in our cases. Some authors consider PSARP only if there was no transillumination of the endoscope light or indentation by Hegar from the rectum to the perineum indicating a long distance between the pouches [3].

In conclusion, transanal end-to-end rectoanal anastomosis allows a safe and an anatomical reconstruction of the anorectum in a significant proportion of patients with rectal atresia, thus avoiding the potential complications associated with the open posterior sagittal approach. However, to achieve an easy and safe anastomosis, a previously inserted low sigmoid colostomy is required. The main strength of our study is that it is the largest study reporting this technique for repair of rectal atresia; nevertheless, a longer period of follow-up is required.

## Data Availability

The datasets used and/or analysed during the current study are available from the corresponding author on reasonable request.
